# Nucleotide Composition and Codon Usage Across Viruses and Their Respective Hosts

**DOI:** 10.3389/fmicb.2021.646300

**Published:** 2021-06-28

**Authors:** Diego Simón, Juan Cristina, Héctor Musto

**Affiliations:** ^1^Laboratorio de Genómica Evolutiva, Departamento de Biología Celular y Molecular, Facultad de Ciencias, Universidad de la República, Montevideo, Uruguay; ^2^Laboratorio de Virología Molecular, Centro de Investigaciones Nucleares, Facultad de Ciencias, Universidad de la Republica, Montevideo, Uruguay; ^3^Laboratorio de Evolución Experimental de Virus, Institut Pasteur de Montevideo, Montevideo, Uruguay

**Keywords:** viral diversity, base composition, GC-content, compositional correlations, codon usage

## Abstract

The genetic material of the three domains of life (Bacteria, Archaea, and Eukaryota) is always double-stranded DNA, and their GC content (molar content of guanine plus cytosine) varies between ≈ 13% and ≈ 75%. Nucleotide composition is the simplest way of characterizing genomes. Despite this simplicity, it has several implications. Indeed, it is the main factor that determines, among other features, dinucleotide frequencies, repeated short DNA sequences, and codon and amino acid usage. Which forces drive this strong variation is still a matter of controversy. For rather obvious reasons, most of the studies concerning this huge variation and its consequences, have been done in free-living organisms. However, no recent comprehensive study of all known viruses has been done (that is, concerning all available sequences). Viruses, by far the most abundant biological entities on Earth, are the causative agents of many diseases. An overview of these entities is important also because their genetic material is not always double-stranded DNA: indeed, certain viruses have as genetic material single-stranded DNA, double-stranded RNA, single-stranded RNA, and/or retro-transcribing. Therefore, one may wonder if what we have learned about the evolution of GC content and its implications in prokaryotes and eukaryotes also applies to viruses. In this contribution, we attempt to describe compositional properties of ∼ 10,000 viral species: base composition (globally and according to Baltimore classification), correlations among non-coding regions and the three codon positions, and the relationship of the nucleotide frequencies and codon usage of viruses with the same feature of their hosts. This allowed us to determine how the base composition of phages strongly correlate with the value of their respective hosts, while eukaryotic viruses do not (with fungi and protists as exceptions). Finally, we discuss some of these results concerning codon usage: reinforcing previous results, we found that phages and hosts exhibit moderate to high correlations, while for eukaryotes and their viruses the correlations are weak or do not exist.

## Introduction

Viruses are obligate parasites of all free cellular life forms and are, at the same time, the most abundant biological entities on Earth (Cobián Güemes et al., 2016). To understand the relationship among different viruses several distinct approaches have been used (Krupovic et al., 2019), given: (i) the diversity of the architecture of their genetic material, which can be DNA or RNA, double-stranded (ds) or single-stranded (ss), linear or circular, segmented or not; (ii) the huge variation of their size (from very tiny particles of around 10 nm with genomes of only a few kb, to giant viruses that reach 1.5 μm and genomes of up to 2.5 Mb that fall into the genome and particle size ranges typical of Bacteria and Archaea); and (iii) since there are not orthologous genes shared by all viruses, it is universally accepted that these biological entities appeared several times in the course of evolution (Koonin et al., 2006; Holmes, 2011; Durzyńska and Goździcka-Józefiak, 2015; Krupovic et al., 2019). Although a lot of work has been done in order to understand the origin and evolution of viruses, and in particular, of their different genetic materials, a complete picture still lacks. One of the simplest approaches for studying organisms and the relationship among them is analyzing the respective “genomic signatures,” which can go from simple base composition as molar content of guanine plus cytosine (GC content), dinucleotides (diNs), and codon and amino acid usage.

Previous phylogenetic studies carried out in different viruses have high-lighted mutational pressure as the major factor in shaping virus evolution in comparison with natural selection (Jenkins and Holmes, 2003; Gu et al., 2004). Nevertheless, as our understanding of virus evolution increases, it appears that although mutational pressure is still a major driving force, it is not the only factor when considering different RNA and DNA viruses (Berkhout and van Hemert, 1994; Chen, 2013; Kustin and Stern, 2021). Moreover, viral genome composition may also be related to virus-host interaction, for instance, by avoiding recognition by the innate immune system (van Hemert et al., 2014). This could provide strong selective pressures, leaving genomic signatures typical of their hosts, both at the nucleotide (Simón et al., 2017) and structural levels (Kindler and Thiel, 2014).

In prokaryotes and eukaryotes, the analyses of these features have led to several conclusions, and perhaps the more relevant for our current purpose can be summarized as follows: (i) base composition is generally more similar within phylogenetically close groups and species living in the same –or very similar– environment (Foerstner et al., 2005; Agashe and Shankar, 2014; Reichenberger et al., 2015), (ii) for prokaryotes, GC content strongly correlates with the mean values for GC1, GC2, and GC3 (that is, the GC content of the three codon positions) for each organism, and also with the global diNs frequencies and amino acid usage (Zhou et al., 2014), (iii) although the variability in genomic GC among prokaryotes is high, within genomes they are remarkably homogeneous (Bohlin and Pettersson, 2019), thought “protoisochores” were found in some Archaea (Khrustalev and Barkovsky, 2011). But on the contrary, (iv) vertebrate genomes (mainly those of mammals and birds) display large contiguous regions characterized by very similar GC content which are termed isochores (Bernardi et al., 1985; Eyre-Walker and Hurst, 2001; Costantini and Musto, 2017), and each of these isochores display a particular and very similar pattern of codon usage (Costantini et al., 2009) and amino acid frequencies (Sabbia et al., 2007), although intragenic GC content heterogeneity has been noted in birds (Khrustalev et al., 2014). Among unicellular eukaryotes, it has been shown that most of them are compositionally heterogeneous (Costantini et al., 2013) as is the case in some flatworms (Lamolle et al., 2016). Therefore, from the study of the genomic composition important features like diNs frequencies and codon usage have been derived, and helped us to understand important biological properties, like patterns of synonymous and non-synonymous substitutions, and the relative effects of neutral and selective forces driving these changes (Pracana et al., 2020).

However, although some recent publications have analyzed several viruses (see, for example, Auewarakul, 2005; Duffy et al., 2008; Mahmoudabadi and Phillips, 2018), an overview focusing on the genomic composition of all viruses is relevant given the impressive increase in viral sequences availability in the last years. In this report, we present the following analyses: (i) base frequencies of all available viruses, (ii) the same feature but sorting viruses according to the Baltimore classification: dsDNA, ssDNA, dsRNA, positive ssRNA (+ssRNA), negative ssRNA (-ssRNA), +ssRNA retro-transcribing (+ssRNA-RT), and dsDNA retro-transcribing (dsDNA-RT), (iii) besides, we analyzed the correlations that hold between the non-coding GC content vs. GC1, GC2 and GC3, (iv) for each group we studied the GC content variation of the viral genomes compared to that of the respective host, and (v) finally, we analyzed codon usage patterns among viruses in relation to the same features of their hosts.

Our main conclusions are that: (i) different viruses (according to the nature and architecture of the respective genetic material), show different properties at their base composition; (ii) there are strong compositional correlations among non-coding regions and the three codon positions; (iii) while GC content of phages strongly correlates with the genomic GC of their hosts, this is not the case for eukaryotic systems; and (iv) in general, the codon usage of phages is dependent of the codon usage of prokaryotes, while the codon usage of animal and plant viruses do not seem to be adapted to the codon usage of their hosts, with the probable exception of fungi and protists.

## Materials and Methods

Sequences were retrieved from NCBI RefSeq viral genomes, Release 205, accessed at ftp://ftp.ncbi.nlm.nih.gov/genomes/refseq/viral/ (Brister et al., 2015). Each viral species was included only once to avoid the overrepresentation of viruses for which there are multiple sequences. For this purpose, only one representative was considered for each viral species (i.e., one representative per taxonomy identifier, TaxID) in this taxonomic rank (*N* = 9,994; see Table 1 and Supplementary Table 1). In the case of segmented viruses, we use global compositional values to summarize these genomes.

**TABLE 1:** The total number of viruses analyzed and within each Baltimore classification group.

Total*	dsDNA	ssDNA	dsRNA	+ssRNA	–ssRNA	+ssRNA-RT	dsDNA-RT
9,994	4,165	1,951	388	1,551	621	78	107

*^∗^The total number (*N* = 9,994) does not match the sum of Baltimore classification groups (*N* = 8,861) because for some viruses the nature of the genetic material and/or strandedness remains unknown.*

Compositional features for non-coding regions and coding GC content per codon position (i.e., GC1, GC2, and GC3), were calculated for genomic regions extracted with BEDTools (Quinlan and Hall, 2010). Host GC contents were scrapped from NCBI Genomes website accessed at https://www.ncbi.nlm.nih.gov/genome (Benson et al., 2017). Codon usage tables were retrieved from HiVE’s CoCoPUTs database (Alexaki et al., 2019).

Virus-host relationships were obtained from Virus-Host Database, accessed at https://www.genome.jp/virushostdb (Mihara et al., 2016). In Table 2 is displayed the diversity of hosts represented in this study; it must be taken into account that the same host will have several viruses assigned to it, while the same virus may be assigned, in some cases, to more than one host. In total, this part of the study included 8,411 host-virus pairs (see Supplementary Material).

**TABLE 2:** The total number of hosts represented in this study and within each taxonomic group considered.

Total	Animals	Archaea	Bacteria	Fungi	Plants	Protists
1,170	378	31	486	72	181	22

The base composition distributions were drawn using kernel density plots with default bandwidths. To test for unimodality/multimodality, Hartigans’ dip tests were performed. The Spearman’s rank correlation coefficient (ρ) was chosen to measure the strength of a linear association between variables. The adjusted R^2^ (adjR^2^) coefficient was used to access the goodness of fit of linear regression models to the data. All these computations were implemented in R v4.0.[0-5] (R Core Team, 2020). Figures were constructed in RStudio v1.3.1073 (RStudio Team., 2020) using RColorBrewer v1.1-2 (Neuwirth, 2014).

## Results

### Base Composition

**FIGURE 1:**
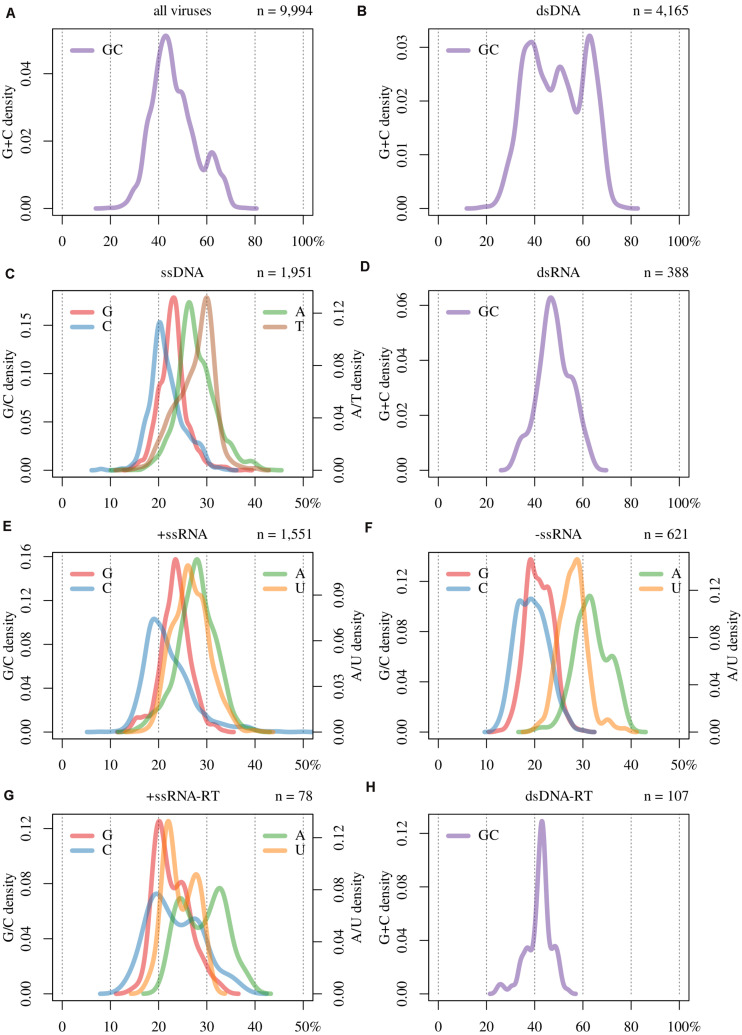
Base composition of **(A)** all viruses, and by Baltimore classification groups **(B–H)**; i.e., **(B)** double-stranded DNA (dsDNA), **(C)** single-stranded DNA (ssDNA), **(D)** double-stranded RNA (dsRNA), **(E)** positive single-stranded RNA (+ssRNA), **(F)** negative single-stranded RNA (-ssRNA), **(G)** +ssRNA retro-transcribing (+ssRNA-RT), and **(H)** dsDNA retro-transcribing (dsDNA-RT).

In Table 1 are displayed the number of all the viral sequences we have analyzed, sorted by Baltimore classification. In Figure 1A is displayed the genomic GC content of all these sequences. It can be seen that the distribution of the genomic GC ranges from 18% to 77%. Furthermore, it is non-unimodal (Hartigans’ dip test, *p*-value = < 0.0001) displaying two modes: a major at a GC of 43% and a minor at 62%. This distribution also presents three shoulders at ≈ 30%, 36%, and 49%, being the latter more evident than the others.

In Figures 1B–H are displayed the base composition (i.e., GC content for ds and nucleotide frequencies for ss) of the viruses studied here, sorted by Baltimore classification. In Figure 1B it can be seen that the GC distribution of dsDNA viruses exhibit a multimodal distribution (Hartigans’ dip test, *p*-value ≈ 0), with three modes at 39%, 51% and 63%. While the value of 39% is representative of the whole sample (see Figure 1A), the other two peaks are due to the overrepresentation of *Escherichia* and *Mycobacterium* bacteriophages. Regarding the range of this distribution, minimum and maximum values were the same for this group as for the complete set of viruses. Thus, the extreme GC values occur within this group.

In Figures 1D,H are plotted the GC content of the other viruses which display double-stranded genomes: dsRNA (Figure 1D) and dsDNA-RT (Figure 1H). The former shows a unimodal distribution with a mode at 46% and displays two shoulders located at GC values of 38% and 58%, respectively. In the case of dsDNA-RT, it shows a symmetrical distribution, peaking at a GC of 43% and with two bumps at 37% and 48%.

The other group of retro-transcribing viruses, +ssRNA-RT, tends to present bimodal distributions in all four bases (Figure 1G), as is the case for GC content (Supplementary Figure 1D; Hartigans’ dip test, *p*-value < 0.01). In Figures 1C,E,F are plotted the remaining single-stranded genomes. Overall, C is the less frequent base, which reflects the process of cytosine deamination which leads to thymine or uracil. This is reinforced by the fact that in ssDNA viruses, T is the most frequent base. In the case of ssRNA viruses, U is the second base in frequency. Furthermore, in these entities, A is the most abundant nucleotide. Taken globally, for all these cases, A and U(T) are the most frequent bases.

### Compositional Correlations

As happens in prokaryotes and most parasitic or symbiotic unicellular eukaryotes, for viruses protein-coding regions make up the majority of their genomes. In summary, only 9% (median) of a viral genome is not transcribed and translated. However, these regions are usually highly structured and encode *cis-*acting elements. Despite this, non-coding and genomic GC display a very high correlation (ρ = 0.86).

**FIGURE 2:**
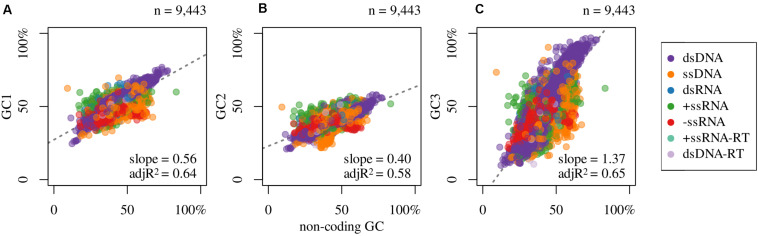
Association between GC content of non-coding regions and **(A)** GC1, **(B)** GC2, and **(C)** GC3, and the slope and adjusted R^2^ (adjR^2^) of the linear regression model (dashed line) for all colored dots (regardless of color).

In Figure 2 are shown the compositional correlations that hold between GC1 (Figure 2A), GC2 (Figure 2B), and GC3 (Figure 2C) with the non-coding GC content of the corresponding virus. These compositional correlations are, in all cases, positive and highly significant (*p*-values ≈ 0). The Spearman correlation coefficients between non-coding GC and GC1, GC2, and GC3 are 0.76, 0.77, and 0.77, respectively. Also, they present big differences in the slopes: 0.57 (GC1), 0.41 (GC2), and 1.37 (GC3). The correlations that hold between non-coding regions and GC1, GC2, and GC3 in viruses sorted by Baltimore classification are displayed in Table 3.

**TABLE 3:** Spearman’s rank correlation coefficients between non-coding regions and GC1, GC2, and GC3, when available within viral genomes, sorted by Baltimore classification group.

Baltimore	GC1	GC2	GC3	n
dsDNA	0.97	0.94	0.94	3,884
ssDNA	0.47	0.56	0.42	1,881
dsRNA	0.57	0.60	0.57	352
+ssRNA	0.50	0.58	0.52	1,486
–ssRNA	0.61	0.54	0.71	597
+ssRNA-RT	0.51	0.60	0.69	76
dsDNA-RT	0.48	0.48	0.65	105

**FIGURE 3:**
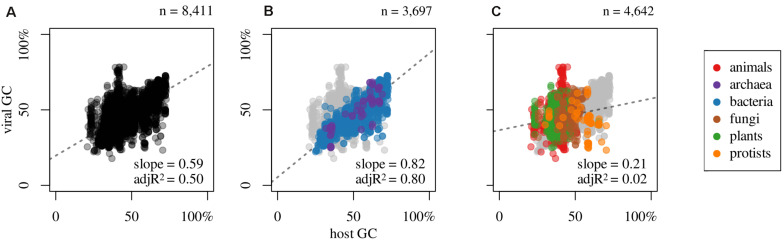
Association between GC content of hosts and that of their infecting viruses for **(A)** all host-virus pairs, **(B)** prokaryotic, and **(C)** eukaryotic, and slope and adjusted R^2^ (adjR^2^) of the linear regression model (dashed line) for all colored dots (regardless of color), omitting pairs that do not apply; i.e., gray dots at the background: **(B)** eukaryotics or **(C)** prokaryotics.

Besides these compositional correlations, inherent to each viral genome, it is of great interest to search for putative dependencies with respect to their hosts. This is displayed in Figure 3A which shows that there is a linear correlation of viral GC content in relation to their respective host genomic GC, with a Spearman correlation coefficient of 0.61. Furthermore, the GC content of phages strongly correlates to their host values; see Figure 3B (ρ = 0.89; *n* = 3,697 host-phage pairs). This holds when considering separately Bacteria (ρ = 0.90, *n* = 3,629) or Archaea (ρ = 0.81, *n* = 68). It is interesting to note that most phages display lower GC values than their hosts. This is noticeable in Figure 3B, since a major proportion of blue and purple dots (prokaryotes) are placed below the 1:1 diagonal.

Contrary to what is provided for prokaryotes, eukaryotic viruses show a very weak correlation between their GC values and that of their hosts; see Figure 3C (ρ = 0.19; *n* = 4,642 host-virus pairs). This figure represents the relationship of eukaryotes and their viruses, colored by eukaryotic subgroup (i.e., animals, plants, fungi, and protists). No meaningful correlation exist between viruses and animals (ρ = 0.14, *n* = 2,691) or plants and their viruses (ρ = 0.09, *n* = 1,672). Conversely, fungi and mycoviruses (i.e., viruses that infect fungi), do present a moderate positive correlation (ρ = 0.43, *n* = 218). Protists and their viruses exhibit a negative correlation (ρ = −0.48, *n* = 61), which, although moderate, is a polarizing result.

### Codon Usage

Given the pattern described above regarding GC content, we further analyzed the relationship between codon usage of viruses in relation to that of their hosts. In Table 4 are displayed the Spearman correlation coefficients for each codon between viruses and hosts. For prokaryotes, all 64 codons show positive correlations between phages and their hosts (ρ values ranging from 0.13 to 0.92) with a median of 0.73, while for eukaryotes the median is 0.08 (ranging from −0.13 to 0.28). Moreover, all but one of the ρ values for phages and their hosts are stronger than any case for eukaryotic system, with the sole exception of the codon CGA (ρ = 0.13). The median adjR^2^ also captures these strong differences between phages and eukaryotic viruses: 0.52, and less than 0.01, respectively.

**TABLE 4:** Spearman’s correlation coefficients (ρ) and adjusted R^2^ (adjR^2^) coefficients between codon frequencies of phages (first and second columns) or eukaryotic viruses (third and fourth columns), and the respective values or their hosts.

	Phages		Eukaryotic viruses	
		
Codon	ρ	adjR^2^	ρ	adjR^2^
UUU	0.85	0.70	0.01	0.00
UUC	0.71	0.50	−0.13	0.00
UUA	0.92	0.85	0.14	0.00
UUG	0.49	0.20	0.14	0.02
CUU	0.64	0.36	0.10	0.01
CUC	0.85	0.65	0.07	0.01
CUA	0.74	0.49	−0.09	0.01
CUG	0.77	0.57	0.19	0.05
AUU	0.82	0.66	0.13	0.01
AUC	0.80	0.62	0.00	0.00
AUA	0.85	0.75	0.06	0.00
AUG	0.60	0.29	0.10	0.00
GUU	0.67	0.46	0.21	0.04
GUC	0.81	0.67	0.05	0.01
GUA	0.77	0.52	−0.00	0.00
GUG	0.75	0.52	0.14	0.02
UAU	0.84	0.69	0.07	0.00
UAC	0.57	0.30	0.13	0.02
UAA	0.73	0.54	0.09	0.00
UAG	0.48	0.13	0.04	0.00
CAU	0.70	0.51	0.19	0.04
CAC	0.83	0.67	−0.03	0.00
CAA	0.87	0.72	0.09	0.00
CAG	0.59	0.47	0.16	0.03
AAU	0.86	0.72	0.23	0.03
AAC	0.26	0.09	0.04	0.00
AAA	0.90	0.82	0.02	0.00
AAG	0.35	0.11	0.23	0.01
GAU	0.73	0.57	0.16	0.03
GAC	0.78	0.65	0.25	0.05
GAA	0.82	0.67	0.06	0.01
GAG	0.72	0.46	−0.02	0.00
UCU	0.61	0.29	0.05	0.00
UCC	0.71	0.44	−0.01	0.00
UCA	0.80	0.59	0.09	0.00
UCG	0.82	0.72	0.25	0.04
CCU	0.55	0.27	−0.03	0.00
CCC	0.84	0.70	0.13	0.02
CCA	0.65	0.35	0.07	0.00
CCG	0.81	0.62	0.05	0.00
ACU	0.69	0.37	−0.06	0.00
ACC	0.85	0.67	0.24	0.05
ACA	0.81	0.72	−0.02	0.00
ACG	0.65	0.41	−0.00	0.00
GCU	0.49	0.19	0.07	0.01
GCC	0.82	0.61	0.08	0.02
GCA	0.51	0.25	−0.04	0.00
GCG	0.76	0.57	0.04	0.01
UGU	0.72	0.49	0.09	0.01
UGC	0.62	0.39	0.08	0.01
UGA	0.66	0.51	−0.00	0.00
UGG	0.39	0.19	0.07	0.00
CGU	0.54	0.28	0.28	0.05
CGC	0.79	0.56	0.12	0.02
CGA	0.13	0.05	−0.01	0.00
CGG	0.84	0.67	−0.02	0.00
AGU	0.82	0.67	0.10	0.01
AGC	0.35	0.14	0.09	0.02
AGA	0.79	0.62	0.04	0.00
AGG	0.33	0.37	0.15	0.02
GGU	0.48	0.20	0.10	0.01
GGC	0.78	0.56	0.21	0.04
GGA	0.55	0.39	−0.07	0.00
GGG	0.59	0.30	0.16	0.02
Median	0.73	0.52	0.08	0.01

*At the bottom of each column, the median value is presented.*

## Discussion

The most basic approach for characterizing genomes is analyzing the genomic base composition. Although the collective distribution (i.e., utilizing all available viral species fully sequenced), shown in Figure 1A, was statistically bimodal, it presents a major mode that is pervasive in the remaining distributions (Figures 1B–H and Supplementary Figure 1). Certainly, this distribution is biased by dsDNA viruses (Figure 1B), which are predominant in the available data set (Table 1), as the more evident shoulder at 49% and the minor mode at 62% are due to the overrepresentation of phages infecting *Escherichia* and *Mycobacterium* genera, respectively. Despite the previous points, we hypothesize that the maximum of the distribution (GC content peaking at 43%) will not change significantly, as will not the minimum and maximum values. We postulate this latter point, given the nature of the genetic code and the correlations that hold between the global GC content and GC1, GC2, and GC3 (see below). Indeed, these two factors impose constraints on codon usage and on the frequencies of the amino acids that can be coded by each virus (Li et al., 2015).

In this study, we have shown that when sorting viruses according to Baltimore classification, several differences among them are apparent. A singular behavior is seen in the case of dsDNA viruses. While unimodal distributions are found in dsRNA and dsDNA-RT (Figure 1D,H), a trimodal distribution is evident for dsDNA viruses (Figure 1B). As shown in Table 1, this group is very numerous, and therefore the distribution shown here is probably robust. This trimodality is due to the adaptation of the GC content between these viruses and their respective hosts. However, we should stress that of the total number of dsDNA viruses studied (4,165), the majority of them (3,778) are phages, which comprises 91% of the total of this group. Therefore, this distribution is directly linked to the adaptation of phages to the GC content of the prokaryotic hosts (see Figure 3B).

The bimodal distributions of bases from +ssRNA-RT (Figure 1G and Supplementary Figure 1D) are intriguing. This was previously observed among members of the family Retroviridae by Berkhout et al. (2002), although with a reduced sample size. This pattern is not due to be single-stranded, since ssDNA, +ssRNA, and –ssRNA viruses (Figure 1C,E,F) display unimodal distributions. One possible explanation is that different +ssRNA-RT viruses are replicated by enzymes that introduce dissimilar mutational biases (Berkhout et al., 2002). To fully understand this point, it is necessary to analyze deeply these viruses and their respective life cycles and enzymes.

We expected that single-stranded (i.e., ssDNA and ssRNAs) viruses should display, on average, remarkably lower G and C frequencies in relation to double-stranded, since ss genomes are prone to mutations toward A and T/U (Lynch, 2007; Long et al., 2018). However, we did not see extreme differences among Baltimore classes, with the exception of dsDNA viruses, but we found that in ssRNA viruses (Figure 1C,E–G and Supplementary Figure 1), always A is the most frequent base followed by U. This is in agreement with a recent study considering a large number of ssRNA viruses (Kustin and Stern, 2021).

Regarding compositional correlations, the main conclusions that can be reached (Figure 2) are the following: (a) As has been known from a long time (for the first reports see: Muto and Osawa, 1987; D’Onofrio et al., 1991), strong correlations do hold in prokaryotes and eukaryotes between the GC content and the corresponding values of the three codon positions. To the best of our knowledge, this is the first time that a similar result is found for all viruses. This implies that despite (i) the different life cycles of each virus, including hosts, (ii) the different enzymes that duplicate each genome, and (iii) their different genetic material, the mutational bias operates in the same direction (toward GC or AT/U) in any given genome. In other words, whatever the cycle of the virus or the genetic material (Table 3), if the replication and/or repair systems are prone to enrich in either GC or AT/U, it does so in the whole genome, irrespective of the region (coding or non-coding).

(b) In spite of the previous point, as happens with prokaryotes and eukaryotes (Muto and Osawa, 1987; D’Onofrio et al., 1991), the strength of this mutational bias is strongly dependent on the codon position. Although the three codon positions increase (or decrease) with the corresponding non-coding sequences, each position changes with different strength: while GC1 shows a moderate increase (Figure 2A), GC3 shows the greatest variation (Figure 2C) while GC2 is the most constrained (Figure 2B). With no doubt, as it is well documented for prokaryotes, where most compositional studied have been done (Zhou et al., 2014), the different behaviors of the three codon positions reflects the structure of the genetic code. Indeed, while any variation in GC2 leads to an amino acid substitution, GC3 is rather free to change since, with the only exceptions of Trp and Met (which, at least in the universal genetic code, are encoded by only one codon each), most changes in GC3 are synonymous; from this point of view GC1 has an intermediate position.

In summary: (i) these correlations, that hold between non-coding and coding regions and their codon positions are indeed universal. (ii) They are independent of the genetic material: indeed, they can be seen not only in prokaryotes and eukaryotes (with dsDNA as genetic material) but in viruses, which as known, can be ss or dsRNA, ss or dsDNA, retrotranscribed or not. They are independent of the (iii) host and of (iv) the replication enzymes. (v) The structure of the genetic code is the main force that imposes limits to the “degree of freedom” of the correlations with the three codon positions. Hypothetically, a steeper slope between the non-coding sequences of viruses with GC2, similar in magnitude to the one found for GC3 (1.37), could cause that some amino acids would not be used (or used at extremely low frequencies) in viruses displaying extremely high (or low) GC content.

The study of GC content of viruses in relation to the GC content of their hosts (eukaryotes and prokaryotes) displays two completely different patterns. While in the majority of eukaryotes (animals and plants) there appears to be no relation (Figure 3B,C), in prokaryotes does exist a strong positive correlation: as the GC content of the host increases, there is an increment in the genomic GC of the respective phages, which was noted previously by Bahir et al. (2009) and Bohlin and Pettersson (2019), among others. Furthermore, as noted by Rocha and Danchin (2002), the GC content of the phages is, in general, lower than that of the respective hosts. However, it is interesting to note that fungi and their viruses do display a moderate positive correlation. Finally, among protists, we note that there is a negative and significant linear correlation between the two mentioned variables. This latter result needs more data to be more accurately portrayed.

Concerning codon usage, we found a similar pattern as in genomic compositional correlations (displayed in Figure 3). Indeed, for a long time, it has been known that in general there is a strong similarity in codon usage between prokaryotes and their phages (Sau et al., 2005; Esposito et al., 2006; Lucks et al., 2008), mainly with dsDNA phages in relation to ssDNA (Chithambaram et al., 2014). The very weak correlation observed for Arg CGA codon (ρ = 0.13) is interesting in light of the fact that this codon is involved in ribosome stalling when appear paired with CCG (i.e., CGA-CCG codon pair) and with another CGA (i.e., CGA-CGA) (Samatova et al., 2021).

However, from Table 4 it is evident that codon usage in eukaryotic viruses is independent of the codon usage of their hosts (see, for instance: Cristina et al., 2015; Castells et al., 2017; Tian et al., 2018; Anwar et al., 2019), although some exceptions do this general rule exist, at least in some unicellular eukaryotes and giant viruses (Michely et al., 2013). This is important given that a codon usage pattern in viruses similar to their hosts could be advantageous for these obligate parasites, since this would allow them to replicate faster and with a lesser extent of errors (Bahir et al., 2009).

This general lack of adaptation might be due to at least three non-mutually exclusive facts. First, most viruses that infect pluricellular species tend to infect specific tissues, where highly specific expressed genes display in turn different codon frequencies [for example, in the case of humans, see TissueCoCoPUTs database (Kames et al., 2020)]. Second, the concept “adaptation” might imply using the less frequent codons in the infected eukaryote, and thus reduce the competition with the more highly expressed host genes, avoiding placing greater stress on the host cell (Chen et al., 2020). Third, the most predominant force shaping codon usage in some eukaryotic viruses could be the mutational bias intrinsic to the enzymes that replicate their genomes. This would lead to very different GC contents and, consequently, different patterns of codon usage, which might, or might not, coincide with that of the host.

## Conclusion

In this study, we have analyzed several compositional properties of nearly 10,000 viral species: genomic base composition (globally and according to Baltimore classification), correlations among non-coding regions and the three codon positions, and the relationship of viral genomic base composition and codon usage with the same feature of their hosts. This allowed us to confirm, with a high number of viruses and hosts, that the genomic base composition and codon usage of phages strongly correlates with the respective values of their hosts. In contrast, as previously but not consensually reported, animal and plant viruses show no correlation between their GC content and that of their hosts. Finally, while all 64 codons show positive correlations between phages and hosts values, in contrast, for eukaryotes and their viruses, overall, the correlations are weak or do not exist.

## Data Availability Statement

The source code and datasets presented here are available on GitHub at: https://github.com/lompa/virushostgc.

## Author Contributions

DS and HM conceived and designed the work and drafted the manuscript. DS conducted all bioinformatics analyses and arranged figures and tables. DS, JC, and HM revised the manuscript, participated in the literature search and discussion, and read and approved the final manuscript. All authors contributed to the article and approved the submitted version.

## Conflict of Interest

The authors declare that the research was conducted in the absence of any commercial or financial relationships that could be construed as a potential conflict of interest.

## Abbreviations

diNs: dinucleotides
ds: double-stranded
dsDNA: double-stranded DNA
dsDNA-RT: double-stranded DNA retro-transcribing
dsRNA: double-stranded RNA
GC: guanine plus cytosine
GC1: guanine plus cytosine content of first codon position
GC2: guanine plus cytosine content of second codon position
GC3: guanine plus cytosine content of third codon position
ρ (rho): Spearman’s correlation coefficient
ss: single-stranded
ssDNA: single-stranded DNA
-ssRNA: negative single-stranded RNA
+ssRNA: positive single-stranded RNA
+ssRNA-RT: positive single-stranded RNA retro-transcribing.
