# Supplementary figures and images for: Nucleotide Composition and Codon Usage Across Viruses and Their Respective Hosts
Source: Front Microbiol. 2021 Jun 28;12:646300. doi: 10.3389/fmicb.2021.646300 (PMC8274242; doi:10.3389/fmicb.2021.646300)

## Slide 1
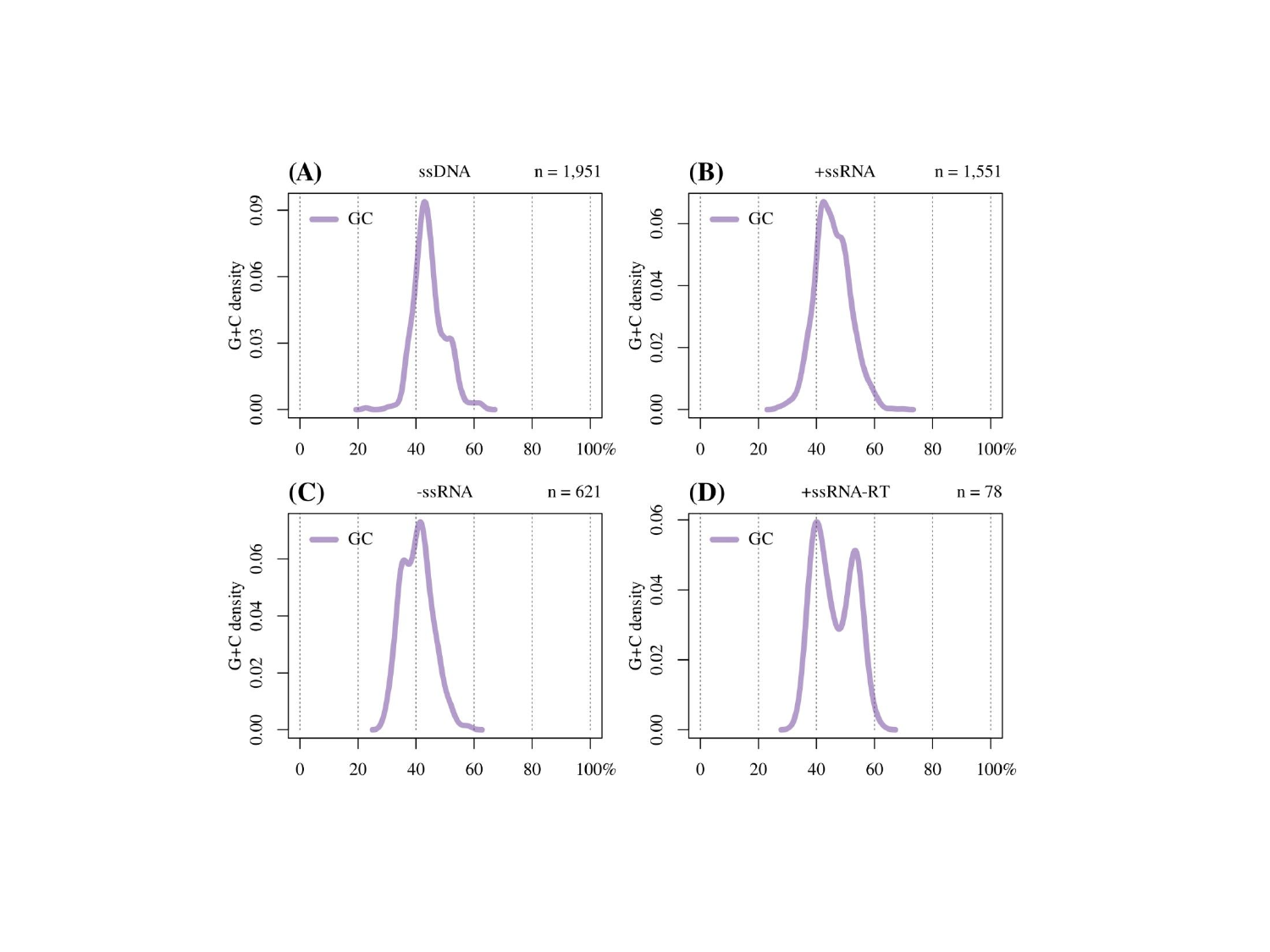

Supplement: Supplementary file 1 [file Presentation_1.PPTX]
